# School-based healthcare services in Cape Town, South Africa: When there’s a will, there’s a way

**DOI:** 10.4102/phcfm.v15i1.4216

**Published:** 2023-10-27

**Authors:** Nadia Ahmed, Carey Pike, Jessica Lee, Colleen Wagner, Linda-Gail Bekker

**Affiliations:** 1 Mortimer Market Centre, Central North West London NHS Trust, London, United Kingdom; 2 Desmond Tutu HIV Centre, Faculty of Health Sciences, University of Cape Town, Cape Town, South Africa; 3 Geisel School of Medicine, Dartmouth College, Hanover, New Hampshire, United States of America; 4 Networking HIV and AIDS Community of South Africa (NACOSA), Cape Town, South Africa

**Keywords:** school health services, adolescent health, HIV prevention, adolescent pregnancy, sexual and reproductive health, South Africa, school health nursing

## Abstract

South African secondary schools do not deliver school-based healthcare services despite high rates of human immunodeficiency virus (HIV) infection, sexually transmitted infections, and unplanned pregnancies among adolescents, ongoing sub-optimal uptake of healthcare services from public healthcare facilities by adolescents, and national policy support for such services. A pilot school health nursing programme (SHNP) was offered to 44 secondary schools in a single health sub-district within the Western Cape, South Africa. The programme included fortnightly nurse visits that offered a standard package of healthcare services, including sexual and reproductive health services tailored according to school preference.

Of the 44 schools, 42 gave permission for the SHNP to operate, with the majority of schools selecting the full comprehensive package of services. Programme implementation was truncated such that delivery only occurred over two school terms (20 weeks); however, 344 students attended the service. The majority of service users were female with a median age of 16 years, and over a half attended the service for sexual and reproductive health services.

**Contribution:**

A key challenge to school-based health service delivery arose from inadequate stakeholder support and differential views of adolescent healthcare needs among government officials, parents, guardians, school staff and governing bodies. These findings motivate for ongoing multi-level stakeholder engagement around the reality of adolescent healthcare needs and further opportunities to deliver school health services for longer time periods such that their feasibility, acceptability, and potential to impact healthcare outcomes can be assessed in this setting.

Adolescents spend approximately one third of their time in school, a setting where healthy lifestyles and healthcare can be promoted and provided through interventions and direct service delivery.^1,2^ School health services are used in 102 countries, through school-based or school-linked provision, with evidence showing that such services provide accessible, efficient, effective and safe management of the healthcare needs of adolescents.^1,2^ South Africa (SA) has a high rate of human immunodeficiency virus (HIV) incidence, sexually transmitted infections (STIs), and unplanned pregnancies among adolescents, indicating unmet health needs by the current facilities and interventions.^3^ While recommended in national policy,^4^ in practice they are not delivered as a standard in South African secondary schools, with limited data to guide effective implementation. Implementation challenges have been attributed to managerial variation in the value attached to health promotion, insufficient collaboration between the departments of health (DOH) and education (DOE), and inadequate stakeholder integration.^5,6^ While deemed acceptable by school staff, previous attempts to implement school health services have been sub-optimal, with infrequent nurse visits (up to once a year), a lack of basic resources (staff, equipment and space), poor referral systems, and greater challenges in rural environments.^4,7^

## Design and delivery

**FIGURE 1:**
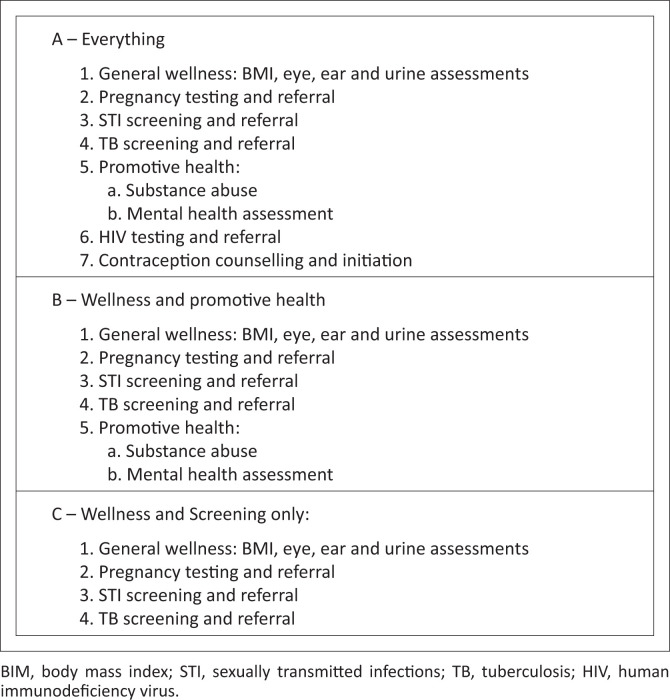
A description of the different levels of service provided in each of the A, B, and C packages of service.

During the implementation of the South African National Young Women and Girls (YWG) Programme, funded by the Global Fund to Fight AIDS, Tuberculosis, and Malaria, a school health nursing programme (SHNP) was offered to 44 secondary schools in a single health sub-district within the Western Cape, SA.^8^ The primary objective was to pilot a potential model of school health delivery to inform the design of future large-scale trial, to provide guidance for implementation. The SHNP involved fortnightly school visits by registered nurses (RNs), offering a range of healthcare services (Figure 1) and urgent advice within school hours. Clinical practice was conducted according to standard practice, with onward referral to other services as needed.^9^ Data on school and learner characteristics were collected routinely as part of the broader YWG programme. Programme records included basic demographics and medical records (reason for visit, test results, administration of referrals).

## Implementation

Of the 45 schools, 42 participated. Reasons for decline amongst the remaining three schools included: commitment to other programmes, local safety concerns, and school governing body opposition. Forty per cent (*n* = 17) of schools opted for package A, 43% (*n* = 18) for B and 17% (*n* = 7) for C. Schools of a lower socioeconomic status, larger size, higher HIV and tuberculosis (TB) burden, tended to opt for package A. Schools with higher socioeconomic status and performance, lower HIV and TB disease burdens but higher pregnancy prevalence and substance use, opted for package B, C, or declined altogether. The SHNP was scheduled for 12 months; however, a communication change from district education officials led to early termination, limiting the delivery period to two of the four school terms (20 weeks).

A total of 344 school learners were seen, and the majority of them were female (*n* = 258, 81%), with a median age of 16 years (IQR 14–17). Over a half attended for sexual reproductive health (SRH) related matters (55%, *n* = 189), including STIs, contraception, menstruation issues and circumcision requests. Fifty per cent (173/344) of the learners received an HIV test; 4% (5/138) had genital symptoms requiring onward referral for management; 9% (24/258) requested pregnancy tests; and 12 males enquired about circumcision following an educational session on HIV prevention. Contraception was used by 16% (41/258) of females, and 61% (131/344) never used condoms. The most common HIV risk factor reported was not knowing their partner’s HIV status. Eight per cent (17/203) of those screened, reported a history of sexual and/or physical abuse. Depression was indicated in 11% of learners through the Patient Health Questionnaire 9 screening tool. Other minor ailments seen required simple reassurance, advice or management.

Thirteen per cent (44/344) in total required onward referral to another service. Healthcare facility referrals were needed for 57% (25/44), social services for 43% (19/44), with the remainder having their presenting complaint resolved on-site.

## Discussion

The key challenge to the SHNP pilot in secondary school health service delivery came from inadequate stakeholder support and differential views of adolescent healthcare needs among government officials, parents, guardians, school staff and governing bodies. The package of healthcare services designed for the pilot, package A, met resistance in a subset of participating schools that necessitated the re-design of the package into two reduced offerings, further evidencing divergent views of needs across schools. Different opinions from district education officials however ultimately led to early termination, despite best efforts to reach effective solutions. The DOE has traditionally worried about activities that are not directly related to education and curricula. It is incumbent upon us to show that benefits outweigh any perceived risks and that this can be performed efficiently, minimising time that learners may take from school-related activities rather than increasing that time.

While the sample size was low overall, this reflects a short pilot period for reasons aforementioned. Furthermore, fortnightly RN visits might have further impacted the low sample size; had the RN visits been more frequent, more learners may have had the opportunity to be seen. Increased familiarity with the service over an extended period could have increased uptake, especially among traditionally difficult-to-engage male users, given that adolescents find services more confidential, reliable and consistent when there is time to develop rapport with healthcare providers.^9,10^

Learner enquiries into circumcision additionally support the ability of the SHNP to provide an immediate link for applying learning into practice. The need for health education and/or empowerment interventions in tandem with health services was further evident from the sexual risk profiles of learners. Risk factors among adolescents are chronically under-reported, for reasons including social desirability bias and confidentiality concerns, and as such the figures reported here are likely underestimates. More time to build rapport and trust with the RNs may have reduced underreporting bias, as well as led to opportunities to manage sexual reproductive health presentations but more importantly screening and prevention of these issues and others.

Approximately one third required external referrals, while the majority had the potential to be dealt with on-site, but was limited by specialisation (e.g. prescribing) and/or permissions (e.g. contraception provision). This supports the potential for school health services to, at a minimum, effectively increase access to healthcare for adolescents and to decongest health facilities.

Despite these challenges and limitations, there were encouraging and frequent reports of positive feedback that the SHNP was acceptable among learners, school staff, local DOE officials, and the RNs. No formal feedback was obtained; however, learners participating in focus group discussions linked to the broader YWG programme evaluation reported finding the SHNP acceptable; ‘We go to the clinic and it’s already packed … here it’s never packed’ and ‘clinic nurses are rude … Here, the nurse doesn’t shout, she just helps’.^8^

Findings from this SHNP provide the beginning of a successful model of care for delivering healthcare in secondary schools in SA. The delivery and implementation successes and challenges further motivate multilevel stakeholder engagement around the reality of adolescent healthcare needs and a potential solution to support and complement existing healthcare through the delivery of school health services. Longer implementation is needed to refine the model, and assess its impact, feasibility and cost-effectiveness.
